# Effects of transcranial static magnetic stimulation over the primary motor cortex on local and network spontaneous electroencephalogram oscillations

**DOI:** 10.1038/s41598-021-87746-2

**Published:** 2021-04-15

**Authors:** Sumiya Shibata, Tatsunori Watanabe, Yoshihiro Yukawa, Masatoshi Minakuchi, Ryota Shimomura, Sachimori Ichimura, Hikari Kirimoto, Tatsuya Mima

**Affiliations:** 1 Kinugasa Research Organization, Ritsumeikan University, 56-1, Tojiin, Kitamachi, Kita-ku, Kyoto, Kyoto 603-8577 Japan; 2 Department of Sensorimotor Neuroscience, Graduate School of Biomedical and Health Sciences, Hiroshima University, 1-2-3 Kasumi, Minami-ku, Hiroshima, Hiroshima 734-8553 Japan; 3 Department of Rehabilitation, Murata Hospital, 4-2-1, Tajima, Ikuno-ku, Osaka, Osaka 544-0011 Japan; 4 The Graduate School of Core Ethics and Frontier Sciences, Ritsumeikan University, 56-1, Tojiin, Kitamachi, Kita-ku, Kyoto, Kyoto 603-8577 Japan

**Keywords:** Motor cortex, Electroencephalography - EEG, Transcranial magnetic stimulation

## Abstract

Transcranial static magnetic stimulation (tSMS) is a novel non-invasive brain stimulation technique that reduces cortical excitability at the stimulation site. We investigated the effects of tSMS over the left primary motor cortex (M1) for 20 min on the local electroencephalogram (EEG) power spectrum and interregional EEG coupling. Twelve right-handed healthy subjects participated in this crossover, double-blind, sham-controlled study. Resting-state EEG data were recorded for 3 min before the intervention and 17 min after the beginning of the intervention. The power spectrum at the left central electrode (C3) and the weighted phase lag index (wPLI) between C3 and the other electrodes was calculated for theta (4–8 Hz), alpha (8–12 Hz), and beta (12–30 Hz) frequencies. The tSMS significantly increased theta power at C3 and the functional coupling in the theta band between C3 and the parietal midline electrodes. The tSMS over the left M1 for 20 min exhibited modulatory effects on local cortical activity and interregional functional coupling in the theta band. The neural oscillations in the theta band may have an important role in the neurophysiological effects induced by tSMS over the frontal cortex.

## Introduction

Transcranial static magnetic stimulation (tSMS) is a novel non-invasive brain stimulation (NIBS) technique. Through the static magnetic fields (SMFs) produced by a strong, compact neodymium magnet placed on the scalp, tSMS can suppress cortical excitability just below the magnet^1–4^. Furthermore, we previously reported the modulation of the intracortical excitability in the primary motor cortex (M1) contralateral to the M1 where the magnet is placed^5^. Since tSMS is not associated with induced electric currents, it does not provoke seizures or tingling sensations. Therefore, tSMS is a safe and low-cost technique for neuromodulation.

Investigating changes in spontaneous electroencephalogram (EEG) activity by tSMS is crucial to better understand the neurophysiological effects of tSMS. Rhythmic brain activity is a fundamental property of neural elements and represents the synchronization of oscillations across them^6^. EEG is an electrophysiological technique that noninvasively records the oscillatory characteristics of neuronal activities. It provides a direct index of neuronal functions with high temporal resolution unlike magnetic resonance imaging (MRI), functional MRI, positron emission tomography, and single-photon emission computed tomography. It can also show clear changes in the synchronization within and among neuronal populations in the cerebral cortex. The synchronization evident in EEG can be revealed through spectral analysis^7^. Changes in the synchronization within the local neuronal populations appear as changes in the EEG power spectrum, while those in the synchronization between the neuronal populations appear like those in interregional EEG coupling^8,9^. Local EEG power spectrum and interregional EEG coupling can be used to assess the neurophysiological effects of tSMS; however, only a limited number of studies have investigated this application^10–12^.

**Figure 1:**
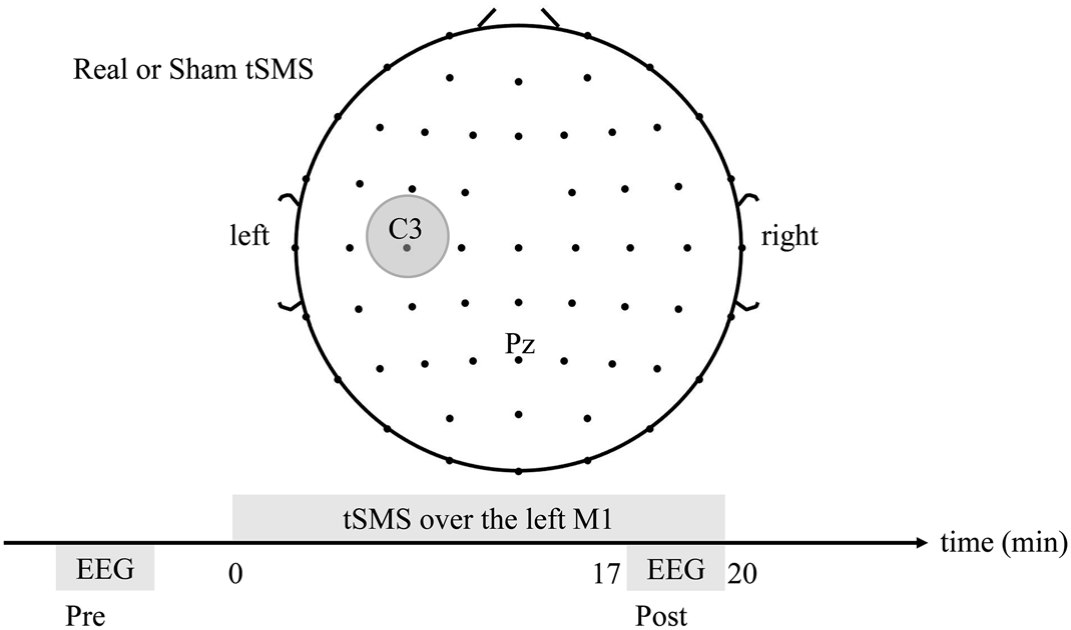
Schematic illustration of the experimental setup. Real or sham tSMS (grey circle) was applied over the left M1 for 20 min. The resting EEG was recorded for 3 min before and during the intervention (Pre and Post respectively).

Other NIBS techniques, that inhibit the cortex function at the stimulation site, can modulate synchronization within and among the neuronal populations in the cerebral cortex^9,13,14^. We hypothesized tSMS to have modulatory effects on both the local EEG power spectrum in the stimulated cortex just below the magnet and the EEG coupling between the stimulated cortex and remote cortex. We conducted a crossover, double-blind, and sham-controlled study to investigate the changes in theta (4–8 Hz), alpha (8–12 Hz), and beta (12–30 Hz) EEG oscillations produced by tSMS. Twelve healthy subjects received real and sham tSMS to their left M1 for 20 min (Fig. 1).

To evaluate the modulation of local cortical activity, the changes in the resting EEG power spectrum at the left central electrode (C3) was compared between the real and sham tSMS. To investigate the modulation of interregional functional coupling, the changes in the weighted phase lag index (wPLI)^15^ between C3 and the other electrodes, was compared between the real and sham tSMS. The wPLI is an index of phase-synchronization between two signals. The range of the wPLI is between 0 and 1. Higher wPLI value indicates higher synchronization of the two signals, and vice versa.

## Results

### Local cortical activity

**Figure 2:**
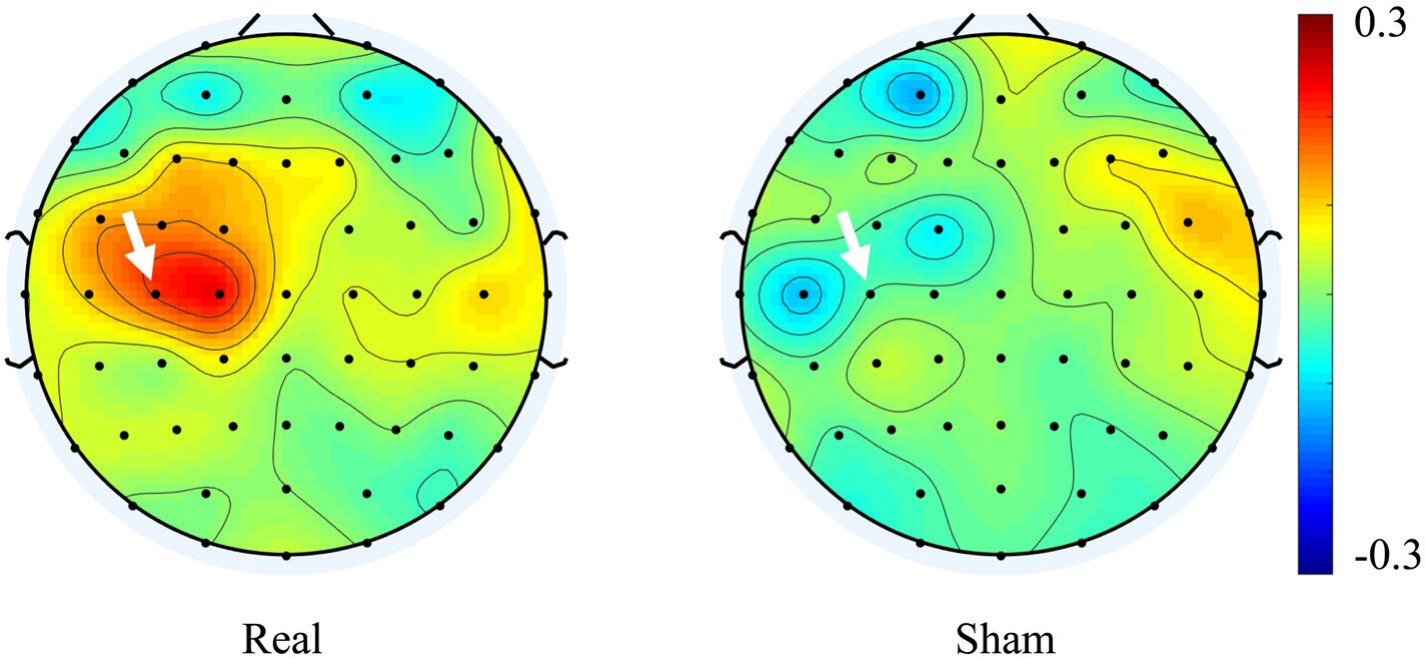
Scalp map of the changes in the EEG power in the theta band averaged across all the subjects for real (left) and sham (right) tSMS. An arrow shows the C3 electrode. Note that the area at C3 was a “hot spot” for real tSMS.

**Figure 3:**
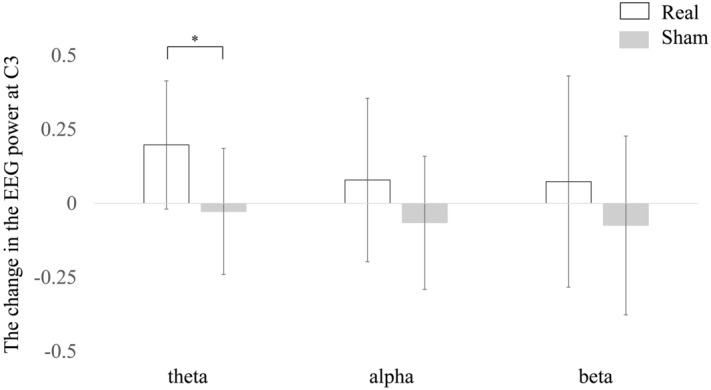
The changes in the EEG power for each frequency band at C3 averaged across all the subjects for the real and sham tSMS. Error bars indicate standard deviation. The change was significant only for the theta band (*).

None of the subjects exhibited any side effects. The changes in the EEG power for the real tSMS and sham tSMS at C3 were 0.20 ± 0.22 and − 0.03 ± 0.21 in the theta band, 0.08 ± 0.28 and − 0.07 ± 0.23 in the alpha band, and 0.07 ± 0.36 and − 0.07 ± 0.30 in the beta band, respectively (the change in the EEG power is a normalized unitless value). Figure 2 shows the changes averaged across all subjects in the theta band for the real and sham tSMS. The area at C3 was a “hot spot” for the real tSMS. The change was significant only in the theta band (*p* = 0.040) (Fig. 3). This demonstrates that tSMS could increase the EEG power in the theta band at the stimulation site.

### Interregional functional coupling

**Figure 4:**
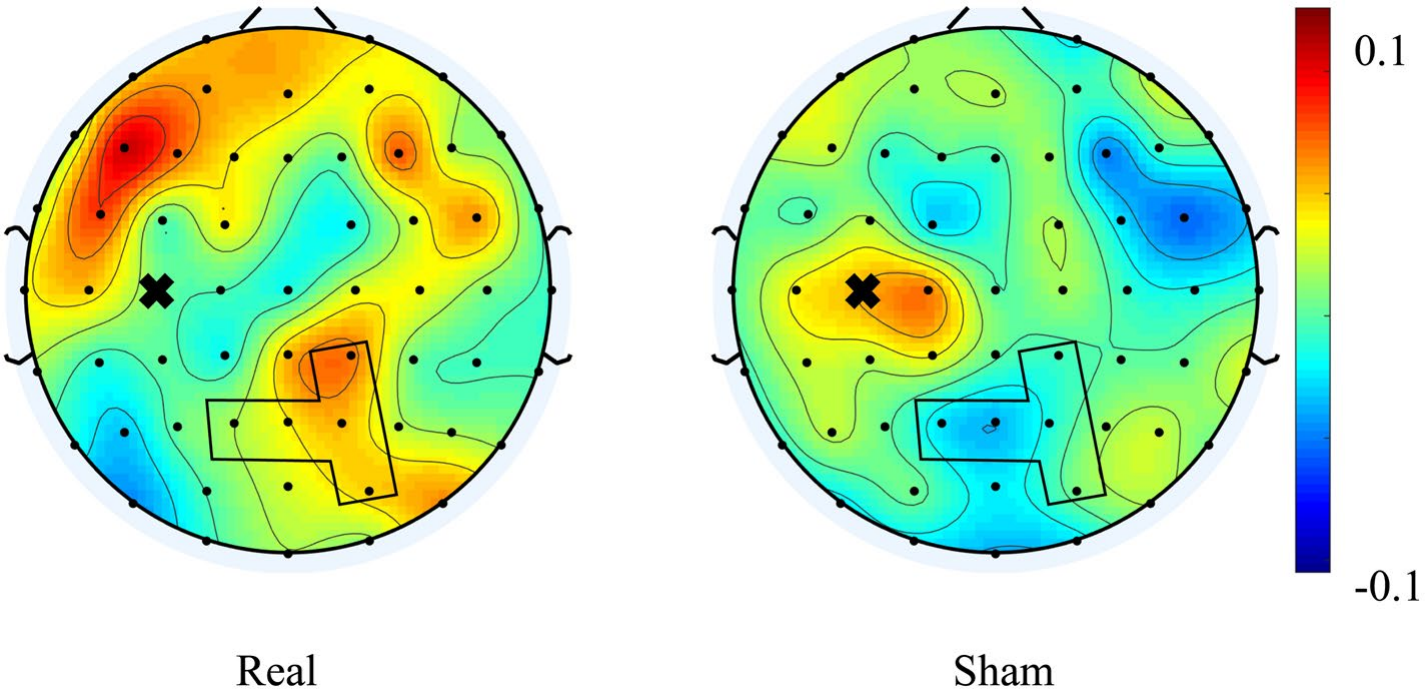
Scalp map of the median across all subjects of the changes in the wPLI in the theta band between C3 and the other electrodes for real (left) and sham (right) tSMS. A cross mark shows the position of C3. The significant cluster was identified at the electrodes surrounded by a black line.

Supplementary Table S1 shows the changes in the wPLI for each frequency band. The largest cluster of significant electrode pairs were identified as C3-Pz [real: 0.04 (0.02–0.08), sham: − 0.04 (− 0.13–0.04)], C3-CP2 [real: 0.07 (− 0.03–0.14), sham: − 0.01 (− 0.04–0.03)], C3-P1 [real: 0.02 (− 0.02–0.13), sham: − 0.03 (− 0.05–0.03)], C3-PO4 [real: 0.05 (0.02–0.08), sham: 0.01 (− 0.06–0.02)], and C3-P2 [real: 0.05 (− 0.01–0.09), sham: − 0.01 (− 0.10–0.03)] in the theta band. The wPLI before and during the intervention for each frequency band is shown in Supplementary Table S2. Figure 4 shows the spatial distribution of the median across all subjects of the changes in the wPLI in the theta band. TSMS could increase the wPLI between the left central area and the parietal midline.

## Discussion

We report that tSMS over the left M1 could increase both the theta EEG power at the stimulation site and functional coupling in the theta band between the stimulation site and the parietal midline. To our knowledge, this is the first study to demonstrate the effects of tSMS on both the local cortical activity and interregional functional coupling.

Regarding the tSMS effect on local cortical activity, previous studies demonstrated that tSMS increased the alpha power on the cortex just below the magnet when applied over the occipital and parietal cortex^10,11^. These studies suggested that an increase in the local alpha oscillations by tSMS reflect the inhibition of visual^10^ and sensory^11^ functions. However, another study showed that tSMS over the dorsolateral prefrontal cortex (DLPFC) did not change the alpha power at the stimulation site^12^. This indicates a location-specific effect of tSMS on local oscillations. In our study, tSMS over the left M1 did not change the alpha power but increased the theta power at the stimulation site. Since tSMS over the DLPFC exhibited a trend-level effect in the theta band^12^, the tSMS over the frontal cortex may modulate the theta oscillations.

TSMS over the left M1 increased the functional coupling between the stimulation site and the parietal midline. We previously demonstrated that tSMS over the M1 has a modulatory effect on the contralateral M1^5^. The present study suggests that the interhemispheric effect of tSMS may result from the modulation of the motor network via the parietal area in the theta band. This speculation is compatible with an MRI study showing that the precuneus was connected to areas implicated in motor execution^16^.

The results of the present study may partly be explained by the neurophysiological effects of tSMS. Although hippocampal theta rhythms in rodents are established, the theta activity can also be recorded from several extrahippocampal regions in rodents and humans^17^. Generators of theta oscillations are likely located near the surface of the brain and widely distributed over the neocortex^18^. Based on the actual measurements of the magnetic field induced by tSMS^4,19^, tSMS had a sufficient SMF to cause a biological effect on the brain surface (an estimated distance of 2–3 cm from the scalp). Therefore, tSMS can have modulatory effects on the theta oscillations generators near the brain surface. Synchronization of cortical theta oscillations over large regions can cause remote effects of tSMS. Although the neurophysiological mechanisms of the tSMS action on the brain are not yet well defined, previous studies reported that tSMS can modulate the inhibitory systems related to GABA receptor activity^20,21^. GABAergic neurons or interneurons are likely involved in hippocampal theta oscillations^22^. The effects of tSMS on theta oscillations may result from the modulation of inhibitory systems related to the GABA receptor activity.

Other NIBS techniques, such as low-frequency repetitive transcranial magnetic stimulation (rTMS) and cathodal transcranial direct current stimulation (tDCS), also inhibit the cortex function at the stimulation site. Variable results exist for the effect of such NIBS techniques on the resting-state EEG power. In healthy humans, low-frequency rTMS (0.9 Hz) over the lateral premotor cortex significantly increased the resting state power in the alpha1 (8–10 Hz) band recorded from the bilateral sensorimotor areas and the mesial frontocentral area, but had no significant effect on the alpha2 (11–13 Hz), beta1 (14–20 Hz) and beta2 (21–30 Hz) bands^9^. In patients suffering from depression, the low-frequency rTMS (1.0 Hz) over the right prefrontal dorsolateral cortex had no significant effect on the resting state power in the delta (1–3.5 Hz), theta (3.5–8 Hz), alpha (8–12 Hz), and beta (12–32 Hz) bands. In healthy humans, the cathodal tDCS over the motor area increased the resting state power in the delta (2–4 Hz) and theta (4–7 Hz) bands^13^. A simultaneous tDCS-EEG study revealed that high-definition cathodal tDCS over the sensorimotor area resulted in a smaller delta (2–4 Hz), theta (4–8 Hz), and alpha (8–13 Hz) response compared to the sham stimulation^14^. Although these differences are likely due to differences in the subject population and stimulation, these previous studies demonstrate that NIBS techniques that inhibit brain functions can also modulate spontaneous brain rhythms.

We show that the neural oscillations in the theta band can be modulated by tSMS over the frontal cortex. Based on these findings, tSMS can be used for clinical application in future. Thalamocortical dysrhythmia, which is an abnormally increased thalamocortical activity in the theta band, is likely related to neurological and psychiatric conditions such as neurogenic pain, tinnitus, Parkinson’s disease, and depression^23^. TSMS may have the potential to modulate such a pathological oscillation in the theta band. Theta activity is also associated with attention and cognitive control in humans and rodents^17^. In humans, the sensory, motor, and cognitive functions related to high gamma activities can be coordinated through the theta/high gamma coupling^24^ indicating that tSMS over the frontal cortex may have behavioral effects through the modulation of theta oscillations. Therefore, tSMS is potentially a safe and low-cost technique for neurological disorders treatment and neurorehabilitation.

The limitation of this study is that EEG does not have a good spatial resolution. Current source density (CSD) estimates through the surface Laplacian computation^25^, used in this study, improve EEG spatial resolution^26^. However, the surface-level connectivity data have spurious false positive connections through field spread in the vicinity of true interactions even when using measures that are immune to zero-lag correlations^27^ such as the wPLI. To avoid neuroanatomical misinterpretations of the coupled sources, it is essential to analyze full source-space interaction mapping. In the cluster-based permutation test for the wPLI, all electrode pairs including C3 were investigated. Considering the result of the cluster-based permutation test for the wPLI, a true interaction was suggested to be localized between the stimulation site and the parietal midline.

In conclusion, we show that tSMS over the left M1 could increase the theta EEG power at the stimulation site and increase the functional coupling in the theta band between the stimulation site and the parietal midline. The oscillatory effects of tSMS may be associated with the modulation of inhibitory systems by the GABA receptor activity. Further studies on the oscillatory effects of tSMS will help elucidate the neurophysiological mechanisms of tSMS.

## Methods

### Subjects

Twelve healthy subjects (23–34 years of age; mean age ± standard deviation, 27 ± 3 years; 7 men) were included in this study. Subjects had no history of neurological illness based on self-report. All subjects were right-handed, as determined by the Edinburgh handedness inventory^28^. The protocol was approved by the Ethics Committees of Ritsumeikan University (Kyoto, Japan) and Murata Hospital (Osaka, Japan). Written informed consent was obtained from all subjects. The study was conducted according to the Declaration of Helsinki.

### Transcranial static magnetic stimulation

A cylindrical nickel-plated (Ni–Cu–Ni) NdFeB magnet with a 50 mm diameter and 30 mm thickness (Model N-50; NeoMag, Chiba, Japan) was used for tSMS. The maximum energy density was 406 kJ/m^3^, with a nominal strength of 863 N. The surface magnetic flux density was approximately 5340 G^5^. A non-magnetic stainless-steel cylinder of similar size and appearance to the real magnet was used for the sham stimulation. The magnet and the non-magnetic cylinder were set using an arm-type light stand (C-stand; Avenger, Cassola, Italy) over the representational area for the right first dorsal interosseous (FDI) muscle (the left M1). They were electrically isolated from EEG electrodes. The left M1 (coil position which led to the largest motor evoked potentials of FDI) was identified by TMS (Magstim 200 magnetic stimulator; Magstim Co., Whitland, UK). The magnet was held tangentially against the subject’s head and the intervention duration was set to 20 min, as with previous studies^5,20^.

### Experimental procedure

Subjects were seated on a chair under normal room light during the experiment. Each subject received both the real and sham tSMS on different days. To avoid the carryover effects^3^, the interval between the real and sham tSMS was set to be more than 3 days. The stimulation performed on the first day was randomly assigned to the subjects; they were blinded to the type of stimulation. EEG data were recorded using a 64-channel electrode cap (EASYCAP, Herrsching, Germany) with electrodes and an EEG amplifier (Brain Products, Gilching, Germany), at a sampling rate of 5 kHz and a 1350 Hz anti-aliasing filter. FCz and AFz were used as the reference and ground, respectively. We maintained an impedance of less than 10 kΩ for each electrode.

Before the intervention, the resting-state EEG data were recorded for 3 min with the eyes open (Pre). The magnet or the sham device was placed on the EEG cap, just above the left M1. Resting-state EEG data were recorded for 3 min with the eyes open 17 min after the beginning of the intervention (Post). During the EEG recordings (Pre and Post), subjects were instructed to fixate on a crosshair at the center of the display, placed 1 m in front of them. The subjects were acoustically isolated through continuous pink noise provided via insert earphones. The testing was performed in a double-blinded manner: Investigator 1 selected and placed the real magnet or sham stainless-steel cylinder, and Investigator 2, who was blinded to the type of intervention being performed, recorded EEGs and analyzed them.

### EEG and statistical analyses

EEG and statistical analyses were performed using EEGLAB^29^, MATLAB (MathWorks Inc., Natick, MA, USA), Excel (Microsoft Inc., Redmond, WA, USA), and SPSS (SPSS Inc., Chicago, IL, USA). Data were down-sampled to 1 kHz and filtered from 1 to 50 Hz. Bad channels were identified through visual inspection and were removed and interpolated. After re-referencing to the common average, data were epoched into 2 s windows. The epochs were visually inspected and the bad epochs containing large artifacts were removed. Independent component analysis (ICA) was conducted using MARA^30^ to remove any remaining artifacts. The resulting data were then converted to CSD^25^ to increase the spatial selectivity.

The theta, alpha, and beta components were obtained by applying the band-pass filters of 4–8, 8–12, and 12–30 Hz to each of the 2 s epoch, respectively. A Hilbert transformation was applied to the filtered signals. To evaluate the effect of tSMS on the EEG power in the cortex just below the magnet, the power spectrum at C3 was analyzed. The EEG activity at C3 represents the primary sensorimotor cortex activity^31^. To evaluate the effect of tSMS on the connectivity between the cortex, just below the magnet, and other areas, the wPLI between C3 and the other electrodes was calculated for each frequency band. The temporal power/wPLI data were calculated for each time point and averaged across all time points over all epochs.

For statistical analysis, the change in the EEG power was computed according to the formula: the change in the EEG power = log_10_ (the EEG power at Post /the EEG power at Pre) (the change in the EEG power is a normalized unitless value). Once the normal distribution was confirmed for the change in the EEG power using the Shapiro–Wilk test (*p* = 0.416), it was compared between the two conditions (real and sham tSMS) using Student’s paired-samples t-test. The result was considered statistically significant at *p* < 0.05.

A non-parametric cluster-based permutation test^32^ was conducted for the analysis of the wPLI between C3 and the other electrodes. The change in the wPLI was computed as the wPLI at Post—the wPLI at Pre. T-values are calculated between the two stimulations for each electrode pair for each frequency band, and 1000 permutations of the two stimulations for each subject were performed to create a distribution of the t-values for each electrode pair for each frequency band. If the original t-value was at the 97.5th percentile on either tail, the electrode pair was considered as significant. If the counterpart electrodes of C3 in two significant electrode pairs were nearest-neighbor (adjacent) electrodes, these electrode pairs were identified as a cluster. The cluster including the largest number of adjacent counterpart electrodes through the three frequency bands was considered as a significant cluster. Values reported in the text are mean ± standard deviation or median (interquartile range).

## Supplementary Information

Supplementary Information

## Data Availability

The datasets generated and/or analyzed during the current study are available from a corresponding author upon reasonable request.
