# Supplementary material for: Effects of transcranial static magnetic stimulation over the primary motor cortex on local and network spontaneous electroencephalogram oscillations
Source: Sci Rep. 2021 Apr 15;11:8261. doi: 10.1038/s41598-021-87746-2 (PMC8050201; doi:10.1038/s41598-021-87746-2)
Supplement: Supplementary file 1 — Supplementary Information [file 41598_2021_87746_MOESM1_ESM.pdf]

Effects of Transcranial Static Magnetic Stimulation over the Primary Motor Cortex on Local and Network Spontaneous Electroencephalogram Oscillations

Sumiya Shibata[1*], Tatsunori Watanabe[2], Yoshihiro Yukawa[3], Masatoshi Minakuchi[3], Ryota Shimomura[3], Sachimori Ichimura[3], Hikari Kirimoto[2], Tatsuya Mima[4*]

1 Kinugasa Research Organization, Ritsumeikan University

56-1, Tojiin, Kitamachi, Kita-ku, Kyoto

603-8577, Kyoto, JAPAN

2 Department of Sensorimotor Neuroscience, Graduate School of Biomedical and Health Sciences, Hiroshima University

1-2-3 Kasumi, Minami-ku, Hiroshima

734-8553, Hiroshima, JAPAN

3 Department of Rehabilitation, Murata Hospital

4-2-1, Tajima, Ikuno-ku, Osaka

544-0011, Osaka, JAPAN

4 The Graduate School of Core Ethics and Frontier Sciences, Ritsumeikan University

56-1, Tojiin, Kitamachi, Kita-ku, Kyoto

603-8577, Kyoto, JAPAN

frequency	region	the changes in the wPLI real	the changes in the wPLI sham	
theta	C3 - Fp1	0.05 (0.02 — 0.09)	0.02 (-0.09 — 0.04)	†
	C3 - Fz	0.02 (-0.03 — 0.08)	-0.01 (-0.03 — 0.04)	
	C3 - F3	0.07 (0.03 — 0.14)	-0.01 (-0.08 — 0.08)	
	C3 - F7	0.02 (0.00 — 0.06)	0.03 (0.01 — 0.07)	
	C3 - FT9	0.00 (-0.04 — 0.13)	0.02 (-0.02 — 0.09)	
	C3 - FC5	0.08 (-0.06 — 0.16)	0.00 (-0.06 — 0.04)	
	C3 - FC1	0.04 (-0.05 — 0.09)	-0.03 (-0.04 — 0.07)	
	C3 - T7	0.04 (-0.02 — 0.12)	0.00 (-0.08 — 0.09)	
	C3 - TP9	0.02 (-0.04 — 0.07)	0.03 (-0.04 — 0.09)	
	C3 - CP5	-0.01 (-0.06 — 0.05)	0.03 (-0.02 — 0.07)	
	C3 - CP1	-0.01 (-0.05 — 0.04)	0.04 (-0.02 — 0.07)	
	C3 - Pz	0.04 (0.02 — 0.08)	-0.04 (-0.13 — 0.04)	* †
	C3 - P3	0.00 (-0.02 — 0.10)	0.00 (-0.03 — 0.03)	
	C3 - P7	-0.02 (-0.07 — 0.02)	-0.01 (-0.04 — 0.03)	
	C3 - O1	-0.01 (-0.14 — 0.03)	-0.02 (-0.09 — 0.00)	
	C3 - Oz	0.02 (-0.03 — 0.08)	-0.04 (-0.06 — 0.02)	
	C3 - O2	0.02 (-0.03 — 0.07)	-0.03 (-0.06 — 0.00)	
	C3 - P4	0.02 (-0.01 — 0.07)	0.01 (-0.07 — 0.04)	
	C3 - P8	0.02 (-0.06 — 0.06)	0.01 (-0.05 — 0.10)	
	C3 - TP10	0.04 (-0.01 — 0.06)	0.03 (0.01 — 0.06)	

C3	-	CP6	0.00	(	-0.08	—	0.02	)	0.00	(	-0.02	—	0.03	)	
C3	-	CP2	0.07	(	-0.03	—	0.14	)	-0.01	(	-0.04	—	0.03	)	* †
C3	-	Cz	-0.01	(	-0.09	—	0.08	)	0.00	(	-0.02	—	0.08	)	
C3	-	C4	0.04	(	-0.02	—	0.08	)	-0.01	(	-0.03	—	0.06	)	
C3	-	T8	-0.01	(	-0.07	—	0.02	)	0.00	(	-0.05	—	0.02	)	
C3	-	FT10	0.02	(	-0.09	—	0.25	)	-0.08	(	-0.11	—	0.00	)	†
C3	-	FC6	0.06	(	-0.04	—	0.10	)	-0.05	(	-0.10	—	0.00	)	
C3	-	FC2	-0.02	(	-0.09	—	0.05	)	0.02	(	-0.05	—	0.05	)	
C3	-	F4	0.07	(	-0.03	—	0.12	)	-0.05	(	-0.10	—	0.02	)	
C3	-	F8	0.01	(	-0.06	—	0.09	)	0.01	(	-0.03	—	0.04	)	
C3	-	Fp2	0.04	(	-0.05	—	0.09	)	-0.02	(	-0.07	—	0.00	)	
C3	-	AF7	0.05	(	0.00	—	0.11	)	0.03	(	-0.03	—	0.10	)	
C3	-	AF3	0.06	(	0.05	—	0.10	)	0.01	(	-0.08	—	0.06	)	
C3	-	AFz	0.05	(	0.00	—	0.11	)	0.02	(	-0.02	—	0.17	)	
C3	-	F1	0.04	(	-0.03	—	0.08	)	0.00	(	-0.06	—	0.09	)	
C3	-	F5	0.11	(	0.01	—	0.15	)	0.03	(	-0.01	—	0.06	)	
C3	-	FT7	0.02	(	-0.06	—	0.19	)	0.00	(	-0.01	—	0.09	)	
C3	-	FC3	0.00	(	-0.03	—	0.07	)	0.02	(	-0.04	—	0.08	)	
C3	-	C1	0.00	(	-0.04	—	0.15	)	0.07	(	0.04	—	0.10	)	
C3	-	C5	0.05	(	0.01	—	0.11	)	0.04	(	0.01	—	0.06	)	
C3	-	TP7	0.01	(	-0.03	—	0.07	)	-0.01	(	-0.07	—	0.05	)	

C3	-	CP3	0.00	(	-0.06	—	0.12	)	0.02	(	-0.01	—	0.05	)	
C3	-	P1	0.02	(	-0.02	—	0.13	)	-0.03	(	-0.05	—	0.03	)	* †
C3	-	P5	-0.03	(	-0.05	—	0.07	)	0.02	(	-0.03	—	0.04	)	
C3	-	PO7	-0.05	(	-0.10	—	-0.02	)	-0.02	(	-0.05	—	0.01	)	
C3	-	PO3	0.00	(	-0.07	—	0.10	)	0.00	(	-0.03	—	0.03	)	
C3	-	POz	0.03	(	-0.01	—	0.09	)	-0.02	(	-0.06	—	0.01	)	
C3	-	PO4	0.05	(	0.02	—	0.08	)	0.01	(	-0.06	—	0.02	)	* †
C3	-	PO8	0.06	(	0.01	—	0.14	)	0.02	(	-0.04	—	0.04	)	
C3	-	P6	0.02	(	-0.02	—	0.05	)	0.02	(	-0.05	—	0.05	)	
C3	-	P2	0.05	(	-0.01	—	0.09	)	-0.01	(	-0.10	—	0.03	)	* †
C3	-	CPz	0.05	(	0.00	—	0.10	)	0.00	(	-0.07	—	0.06	)	
C3	-	CP4	0.01	(	-0.04	—	0.09	)	0.00	(	-0.04	—	0.02	)	
C3	-	TP8	0.00	(	-0.02	—	0.03	)	0.03	(	-0.04	—	0.06	)	
C3	-	C6	0.01	(	-0.01	—	0.05	)	-0.02	(	-0.08	—	0.04	)	
C3	-	C2	0.03	(	-0.03	—	0.11	)	0.02	(	-0.06	—	0.07	)	
C3	-	FC4	0.03	(	-0.07	—	0.10	)	-0.03	(	-0.07	—	0.06	)	
C3	-	FT8	0.00	(	-0.06	—	0.09	)	-0.04	(	-0.08	—	-0.02	)	
C3	-	F6	0.02	(	0.00	—	0.04	)	-0.01	(	-0.09	—	0.10	)	
C3	-	AF8	0.03	(	-0.01	—	0.06	)	0.02	(	-0.08	—	0.10	)	
C3	-	AF4	0.03	(	0.00	—	0.10	)	0.00	(	-0.03	—	0.04	)	
C3	-	F2	0.01	(	-0.04	—	0.05	)	0.01	(	-0.05	—	0.03	)	

	C3	-	Fp1	-0.02	(	-0.10	—	0.02	)	-0.01	(	-0.06	—	0.04	)	
	C3	-	Fz	0.00	(	-0.11	—	0.08	)	0.09	(	-0.03	—	0.13	)	
	C3	-	F3	0.04	(	0.00	—	0.16	)	0.06	(	0.02	—	0.09	)	
	C3	-	F7	-0.08	(	-0.12	—	0.09	)	0.01	(	-0.07	—	0.09	)	
	C3	-	FT9	-0.03	(	-0.12	—	0.04	)	0.01	(	-0.04	—	0.10	)	
	C3	-	FC5	0.01	(	-0.01	—	0.07	)	0.04	(	-0.01	—	0.15	)	
	C3	-	FC1	0.05	(	-0.09	—	0.11	)	0.05	(	0.00	—	0.24	)	
	C3	-	T7	0.06	(	-0.01	—	0.14	)	-0.01	(	-0.09	—	0.05	)	
	C3	-	TP9	-0.01	(	-0.05	—	0.09	)	-0.07	(	-0.11	—	0.03	)	
	C3	-	CP5	-0.04	(	-0.10	—	0.09	)	0.01	(	-0.06	—	0.12	)	
alpha	C3	-	CP1	0.04	(	-0.13	—	0.06	)	-0.03	(	-0.12	—	0.06	)	
	C3	-	Pz	-0.05	(	-0.12	—	0.00	)	-0.03	(	-0.10	—	0.00	)	
	C3	-	P3	-0.08	(	-0.13	—	0.04	)	0.01	(	-0.08	—	0.18	)	
	C3	-	P7	0.06	(	-0.07	—	0.15	)	0.10	(	-0.01	—	0.16	)	
	C3	-	O1	0.00	(	-0.04	—	0.10	)	-0.02	(	-0.04	—	0.05	)	
	C3	-	Oz	-0.06	(	-0.14	—	0.03	)	0.00	(	-0.05	—	0.03	)	
	C3	-	O2	0.00	(	-0.11	—	0.05	)	0.05	(	-0.01	—	0.09	)	
	C3	-	P4	-0.02	(	-0.13	—	0.00	)	0.00	(	-0.11	—	0.12	)	
	C3	-	P8	-0.01	(	-0.06	—	0.01	)	0.07	(	0.01	—	0.19	)	†
	C3	-	TP10	0.00	(	-0.15	—	0.06	)	0.02	(	-0.02	—	0.15	)	

C3	-	CP6	-0.04	(	-0.11	—	-0.02	)	0.05	(	-0.01	—	0.19	)	†
C3	-	CP2	-0.02	(	-0.09	—	0.09	)	-0.06	(	-0.14	—	0.00	)	
C3	-	Cz	-0.04	(	-0.13	—	0.06	)	0.09	(	0.02	—	0.24	)	†
C3	-	C4	-0.01	(	-0.07	—	0.07	)	0.00	(	-0.07	—	0.02	)	
C3	-	T8	-0.04	(	-0.12	—	0.03	)	0.09	(	-0.03	—	0.15	)	†
C3	-	FT10	-0.02	(	-0.05	—	0.13	)	-0.01	(	-0.13	—	0.15	)	
C3	-	FC6	0.02	(	-0.06	—	0.06	)	0.05	(	-0.04	—	0.07	)	
C3	-	FC2	0.06	(	0.01	—	0.08	)	0.01	(	-0.09	—	0.11	)	
C3	-	F4	0.01	(	-0.06	—	0.06	)	0.01	(	-0.04	—	0.11	)	
C3	-	F8	0.07	(	0.02	—	0.13	)	0.05	(	0.00	—	0.16	)	
C3	-	Fp2	-0.07	(	-0.15	—	0.08	)	0.05	(	-0.08	—	0.15	)	
C3	-	AF7	-0.02	(	-0.09	—	0.11	)	-0.01	(	-0.10	—	0.05	)	
C3	-	AF3	-0.01	(	-0.10	—	0.09	)	0.07	(	-0.02	—	0.15	)	
C3	-	AFz	0.05	(	-0.11	—	0.13	)	0.04	(	-0.01	—	0.10	)	
C3	-	F1	-0.04	(	-0.13	—	0.14	)	0.04	(	-0.03	—	0.11	)	
C3	-	F5	0.08	(	-0.05	—	0.17	)	0.04	(	-0.06	—	0.09	)	
C3	-	FT7	-0.07	(	-0.10	—	0.04	)	0.03	(	-0.04	—	0.05	)	
C3	-	FC3	0.09	(	-0.01	—	0.21	)	0.06	(	-0.01	—	0.15	)	
C3	-	C1	-0.03	(	-0.18	—	0.15	)	0.09	(	0.00	—	0.22	)	
C3	-	C5	-0.02	(	-0.12	—	0.08	)	0.01	(	-0.08	—	0.12	)	
C3	-	TP7	0.07	(	-0.01	—	0.15	)	-0.03	(	-0.13	—	0.08	)	

C3	-	CP3	-0.03	(	-0.12	—	0.04	)	0.00	(	-0.08	—	0.16	)	
C3	-	P1	-0.05	(	-0.10	—	0.05	)	-0.02	(	-0.11	—	0.13	)	
C3	-	P5	-0.01	(	-0.09	—	0.05	)	0.06	(	-0.07	—	0.17	)	
C3	-	PO7	0.07	(	-0.04	—	0.09	)	0.03	(	-0.05	—	0.10	)	
C3	-	PO3	0.00	(	-0.07	—	0.08	)	-0.03	(	-0.04	—	0.11	)	
C3	-	POz	-0.06	(	-0.12	—	0.01	)	-0.05	(	-0.07	—	-0.01	)	
C3	-	PO4	-0.07	(	-0.16	—	0.02	)	0.02	(	-0.04	—	0.06	)	
C3	-	PO8	0.00	(	-0.11	—	0.02	)	0.04	(	-0.06	—	0.13	)	†
C3	-	P6	-0.07	(	-0.11	—	0.01	)	0.13	(	-0.05	—	0.23	)	†
C3	-	P2	-0.05	(	-0.09	—	0.05	)	-0.02	(	-0.07	—	0.01	)	
C3	-	CPz	-0.01	(	-0.10	—	0.03	)	-0.02	(	-0.12	—	0.02	)	
C3	-	CP4	-0.09	(	-0.15	—	-0.04	)	-0.05	(	-0.15	—	0.04	)	
C3	-	TP8	0.04	(	0.02	—	0.07	)	0.10	(	-0.04	—	0.25	)	
C3	-	C6	-0.03	(	-0.06	—	0.08	)	0.00	(	-0.05	—	0.09	)	
C3	-	C2	0.02	(	-0.06	—	0.07	)	0.05	(	0.01	—	0.10	)	
C3	-	FC4	0.01	(	-0.01	—	0.05	)	0.02	(	-0.10	—	0.10	)	
C3	-	FT8	0.01	(	-0.01	—	0.07	)	0.04	(	-0.08	—	0.14	)	
C3	-	F6	-0.01	(	-0.05	—	0.05	)	0.02	(	-0.01	—	0.07	)	
C3	-	AF8	-0.03	(	-0.11	—	0.08	)	0.08	(	-0.04	—	0.16	)	
C3	-	AF4	0.02	(	-0.07	—	0.11	)	0.13	(	-0.03	—	0.19	)	
C3	-	F2	0.00	(	-0.07	—	0.10	)	0.04	(	0.02	—	0.09	)	

	C3	-	Fp1	-0.01	(	-0.03	—	0.03	)	0.00	(	-0.04	—	0.02	)	
	C3	-	Fz	-0.03	(	-0.05	—	0.00	)	-0.01	(	-0.04	—	0.03	)	
	C3	-	F3	0.01	(	-0.01	—	0.03	)	0.03	(	0.00	—	0.07	)	†
	C3	-	F7	0.00	(	-0.03	—	0.02	)	0.02	(	-0.03	—	0.06	)	
	C3	-	FT9	-0.01	(	-0.02	—	0.01	)	0.01	(	-0.01	—	0.07	)	†
	C3	-	FC5	0.03	(	0.01	—	0.04	)	0.02	(	-0.02	—	0.06	)	
	C3	-	FC1	-0.01	(	-0.05	—	0.04	)	0.02	(	-0.07	—	0.07	)	
	C3	-	T7	-0.01	(	-0.07	—	0.07	)	0.00	(	-0.04	—	0.04	)	
	C3	-	TP9	0.02	(	-0.02	—	0.05	)	0.01	(	-0.04	—	0.04	)	
	C3	-	CP5	-0.01	(	-0.13	—	0.01	)	0.02	(	0.00	—	0.07	)	†
beta	C3	-	CP1	-0.01	(	-0.07	—	0.03	)	0.01	(	-0.03	—	0.03	)	
	C3	-	Pz	0.00	(	-0.04	—	0.05	)	0.00	(	-0.06	—	0.01	)	
	C3	-	P3	-0.02	(	-0.06	—	-0.01	)	0.00	(	-0.04	—	0.10	)	
	C3	-	P7	-0.05	(	-0.08	—	-0.03	)	0.04	(	-0.01	—	0.07	)	†
	C3	-	O1	0.01	(	-0.03	—	0.03	)	0.00	(	-0.04	—	0.02	)	
	C3	-	Oz	0.00	(	-0.03	—	0.02	)	0.01	(	-0.02	—	0.02	)	
	C3	-	O2	0.00	(	-0.01	—	0.03	)	-0.03	(	-0.05	—	0.01	)	
	C3	-	P4	0.00	(	-0.02	—	0.03	)	0.01	(	-0.01	—	0.02	)	
	C3	-	P8	0.00	(	-0.05	—	0.02	)	0.00	(	-0.02	—	0.02	)	
	C3	-	TP10	0.00	(	-0.01	—	0.02	)	-0.01	(	-0.05	—	0.01	)	

C3	-	CP6	0.01	(	0.00	—	0.03	)	0.00	(	-0.02	—	0.06	)	
C3	-	CP2	0.01	(	-0.01	—	0.02	)	0.01	(	-0.01	—	0.06	)	
C3	-	Cz	-0.01	(	-0.02	—	0.03	)	-0.01	(	-0.03	—	0.05	)	
C3	-	C4	-0.01	(	-0.02	—	0.03	)	-0.01	(	-0.03	—	0.02	)	
C3	-	T8	0.01	(	-0.01	—	0.02	)	-0.01	(	-0.03	—	0.02	)	
C3	-	FT10	0.00	(	-0.02	—	0.03	)	0.02	(	0.00	—	0.04	)	
C3	-	FC6	0.01	(	-0.03	—	0.03	)	-0.01	(	-0.02	—	0.02	)	
C3	-	FC2	0.00	(	-0.02	—	0.02	)	-0.02	(	-0.03	—	0.03	)	
C3	-	F4	0.00	(	-0.05	—	0.02	)	0.04	(	-0.01	—	0.08	)	†
C3	-	F8	0.00	(	-0.05	—	0.02	)	0.01	(	-0.01	—	0.02	)	
C3	-	Fp2	-0.01	(	-0.03	—	0.02	)	-0.01	(	-0.04	—	0.06	)	
C3	-	AF7	0.00	(	-0.02	—	0.04	)	0.00	(	-0.03	—	0.04	)	
C3	-	AF3	-0.01	(	-0.06	—	0.01	)	0.03	(	0.00	—	0.05	)	
C3	-	AFz	-0.01	(	-0.03	—	0.01	)	0.00	(	-0.02	—	0.04	)	
C3	-	F1	-0.02	(	-0.05	—	0.00	)	0.02	(	-0.08	—	0.05	)	
C3	-	F5	0.01	(	-0.02	—	0.04	)	0.03	(	0.00	—	0.09	)	
C3	-	FT7	-0.03	(	-0.06	—	0.02	)	0.01	(	-0.04	—	0.02	)	
C3	-	FC3	-0.02	(	-0.05	—	0.04	)	0.02	(	0.00	—	0.07	)	
C3	-	C1	-0.02	(	-0.04	—	0.06	)	0.02	(	-0.02	—	0.04	)	
C3	-	C5	0.01	(	-0.05	—	0.04	)	0.00	(	-0.01	—	0.05	)	
C3	-	TP7	-0.01	(	-0.03	—	0.01	)	0.01	(	-0.02	—	0.06	)	

C3	-	CP3	-0.02	(	-0.04	—	0.03	)	-0.03	(	-0.04	—	0.06	)	
C3	-	P1	0.00	(	-0.04	—	0.01	)	0.01	(	-0.02	—	0.02	)	
C3	-	P5	-0.04	(	-0.06	—	-0.01	)	0.04	(	-0.03	—	0.10	)	†
C3	-	PO7	-0.04	(	-0.07	—	0.02	)	-0.02	(	-0.03	—	0.03	)	
C3	-	PO3	-0.01	(	-0.03	—	0.00	)	0.00	(	-0.06	—	0.01	)	
C3	-	POz	0.00	(	-0.02	—	0.02	)	-0.01	(	-0.02	—	0.00	)	
C3	-	PO4	0.01	(	0.00	—	0.02	)	-0.02	(	-0.03	—	0.01	)	
C3	-	PO8	0.00	(	-0.01	—	0.02	)	-0.01	(	-0.03	—	0.01	)	
C3	-	P6	0.00	(	-0.01	—	0.03	)	-0.02	(	-0.05	—	0.00	)	
C3	-	P2	-0.02	(	-0.04	—	0.01	)	0.00	(	-0.02	—	0.01	)	
C3	-	CPz	0.02	(	0.00	—	0.04	)	0.00	(	-0.02	—	0.03	)	
C3	-	CP4	0.03	(	0.01	—	0.04	)	0.01	(	-0.05	—	0.05	)	
C3	-	TP8	0.02	(	-0.03	—	0.04	)	0.02	(	-0.03	—	0.05	)	
C3	-	C6	-0.01	(	-0.02	—	0.03	)	-0.01	(	-0.03	—	0.04	)	
C3	-	C2	0.01	(	-0.02	—	0.02	)	-0.01	(	-0.02	—	0.05	)	
C3	-	FC4	0.01	(	-0.02	—	0.03	)	0.01	(	-0.01	—	0.03	)	
C3	-	FT8	0.00	(	-0.03	—	0.01	)	0.00	(	-0.01	—	0.01	)	
C3	-	F6	-0.02	(	-0.05	—	0.02	)	0.01	(	-0.01	—	0.05	)	
C3	-	AF8	-0.04	(	-0.06	—	0.00	)	0.01	(	-0.02	—	0.02	)	
C3	-	AF4	-0.03	(	-0.04	—	0.00	)	0.01	(	-0.02	—	0.03	)	†
C3	-	F2	-0.02	(	-0.04	—	0.00	)	0.01	(	-0.02	—	0.06	)	

Supplementary Table S1

The changes in the wPLI for real and sham tSMS for each frequency band. The largest cluster of significant electrode pairs was identified as C3-Pz, C3-CP2, C3-P1, C3-PO4, and C3-P2 for the theta band (*). The significant electrode pairs are indicated by †.

frequency	region	the wPLI real pre	the wPLI real post	the wPLI sham pre	the wPLI sham post
theta	C3 - Fp1	0.05 (0.03 – 0.11)	0.12 (0.09 – 0.18)	0.09 (0.06 – 0.16)	0.06 (0.03 – 0.12)
	C3 - Fz	0.10 (0.06 – 0.19)	0.15 (0.08 – 0.22)	0.10 (0.07 – 0.20)	0.10 (0.07 – 0.25)
	C3 - F3	0.08 (0.03 – 0.20)	0.17 (0.11 – 0.22)	0.20 (0.08 – 0.25)	0.10 (0.07 – 0.24)
	C3 - F7	0.11 (0.05 – 0.14)	0.13 (0.07 – 0.20)	0.06 (0.01 – 0.11)	0.11 (0.03 – 0.18)
	C3 - FT9	0.06 (0.06 – 0.11)	0.12 (0.03 – 0.26)	0.07 (0.02 – 0.14)	0.06 (0.03 – 0.15)
	C3 - FC5	0.08 (0.05 – 0.19)	0.12 (0.05 – 0.28)	0.10 (0.02 – 0.13)	0.08 (0.05 – 0.11)
	C3 - FC1	0.12 (0.09 – 0.24)	0.16 (0.10 – 0.29)	0.16 (0.07 – 0.18)	0.15 (0.10 – 0.22)
	C3 - T7	0.06 (0.02 – 0.08)	0.08 (0.05 – 0.15)	0.10 (0.07 – 0.13)	0.11 (0.04 – 0.14)
	C3 - TP9	0.08 (0.05 – 0.18)	0.07 (0.03 – 0.23)	0.06 (0.03 – 0.09)	0.11 (0.04 – 0.14)
	C3 - CP5	0.08 (0.04 – 0.15)	0.05 (0.02 – 0.16)	0.05 (0.04 – 0.10)	0.10 (0.07 – 0.12)
	C3 - CP1	0.09 (0.05 – 0.13)	0.06 (0.02 – 0.14)	0.07 (0.02 – 0.12)	0.11 (0.07 – 0.21)
	C3 - Pz	0.03 (0.01 – 0.11)	0.11 (0.07 – 0.15)	0.11 (0.05 – 0.16)	0.07 (0.02 – 0.10)

C3 - P3	0.07 (0.03 – 0.09)	0.06 (0.04 – 0.14)	0.13 (0.05 – 0.18)	0.07 (0.03 – 0.17)
C3 - P7	0.10 (0.03 – 0.15)	0.09 (0.03 – 0.13)	0.09 (0.07 – 0.11)	0.08 (0.06 – 0.09)
C3 - O1	0.10 (0.04 – 0.16)	0.04 (0.03 – 0.08)	0.08 (0.03 – 0.15)	0.05 (0.02 – 0.09)
C3 - Oz	0.05 (0.02 – 0.12)	0.06 (0.04 – 0.12)	0.09 (0.04 – 0.12)	0.04 (0.02 – 0.07)
C3 - O2	0.07 (0.03 – 0.10)	0.09 (0.02 – 0.14)	0.05 (0.03 – 0.11)	0.02 (0.01 – 0.05)
C3 - P4	0.03 (0.02 – 0.11)	0.10 (0.04 – 0.12)	0.10 (0.04 – 0.15)	0.06 (0.04 – 0.15)
C3 - P8	0.06 (0.05 – 0.15)	0.08 (0.03 – 0.16)	0.07 (0.02 – 0.11)	0.06 (0.04 – 0.14)
C3 - TP10	0.09 (0.05 – 0.15)	0.09 (0.04 – 0.23)	0.07 (0.03 – 0.09)	0.09 (0.07 – 0.11)
C3 - CP6	0.09 (0.04 – 0.12)	0.08 (0.02 – 0.13)	0.07 (0.05 – 0.12)	0.06 (0.04 – 0.12)
C3 - CP2	0.07 (0.02 – 0.10)	0.13 (0.04 – 0.18)	0.06 (0.02 – 0.15)	0.09 (0.04 – 0.11)
C3 - Cz	0.07 (0.03 – 0.11)	0.08 (0.04 – 0.14)	0.06 (0.03 – 0.11)	0.09 (0.04 – 0.15)
C3 - C4	0.04 (0.02 – 0.10)	0.08 (0.03 – 0.17)	0.05 (0.02 – 0.11)	0.06 (0.05 – 0.09)
C3 - T8	0.09 (0.04 – 0.12)	0.06 (0.03 – 0.10)	0.05 (0.00 – 0.15)	0.05 (0.03 – 0.09)
C3 - FT10	0.10 (0.04 – 0.15)	0.11 (0.05 – 0.26)	0.13 (0.08 – 0.15)	0.03 (0.02 – 0.06)

C3	-	FC6	0.07	(	0.02	–	0.14	)	0.10	(	0.06	–	0.14	)	0.11	(	0.08	–	0.17	)	0.07	(	0.05 – 0.09)
C3	-	FC2	0.10	(	0.06	–	0.14	)	0.07	(	0.06	–	0.13	)	0.08	(	0.04	–	0.15	)	0.10	(	0.06 – 0.13)
C3	-	F4	0.06	(	0.03	–	0.10	)	0.11	(	0.08	–	0.21	)	0.14	(	0.10	–	0.18	)	0.11	(	0.04 – 0.16)
C3	-	F8	0.09	(	0.04	–	0.15	)	0.11	(	0.09	–	0.14	)	0.07	(	0.03	–	0.17	)	0.07	(	0.01 – 0.20)
C3	-	Fp2	0.05	(	0.03	–	0.08	)	0.07	(	0.04	–	0.13	)	0.07	(	0.05	–	0.13	)	0.06	(	0.04 – 0.09)
C3	-	AF7	0.06	(	0.03	–	0.08	)	0.11	(	0.04	–	0.19	)	0.05	(	0.04	–	0.06	)	0.09	(	0.03 – 0.15)
C3	-	AF3	0.04	(	0.02	–	0.08	)	0.12	(	0.08	–	0.22	)	0.11	(	0.03	–	0.16	)	0.05	(	0.03 – 0.11)
C3	-	AFz	0.08	(	0.03	–	0.10	)	0.15	(	0.07	–	0.21	)	0.05	(	0.02	–	0.12	)	0.09	(	0.02 – 0.22)
C3	-	F1	0.07	(	0.03	–	0.22	)	0.13	(	0.10	–	0.20	)	0.14	(	0.10	–	0.20	)	0.15	(	0.08 – 0.22)
C3	-	F5	0.11	(	0.02	–	0.14	)	0.17	(	0.10	–	0.26	)	0.17	(	0.09	–	0.22	)	0.13	(	0.08 – 0.24)
C3	-	FT7	0.07	(	0.03	–	0.13	)	0.12	(	0.04	–	0.20	)	0.05	(	0.01	–	0.08	)	0.09	(	0.04 – 0.13)
C3	-	FC3	0.09	(	0.09	–	0.22	)	0.16	(	0.07	–	0.22	)	0.10	(	0.07	–	0.21	)	0.11	(	0.08 – 0.21)
C3	-	C1	0.07	(	0.03	–	0.10	)	0.10	(	0.05	–	0.29	)	0.04	(	0.02	–	0.07	)	0.14	(	0.05 – 0.16)
C3	-	C5	0.06	(	0.03	–	0.11	)	0.09	(	0.06	–	0.21	)	0.10	(	0.02	–	0.17	)	0.10	(	0.06 – 0.19)

C3	-	TP7	0.08 (0.05 – 0.13)	0.08 (0.05 – 0.14)	0.10 (0.02 – 0.16)	0.07 (0.05 – 0.10)
C3	-	CP3	0.08 (0.06 – 0.11)	0.08 (0.04 – 0.19)	0.06 (0.04 – 0.13)	0.09 (0.07 – 0.13)
C3	-	P1	0.04 (0.01 – 0.10)	0.09 (0.05 – 0.19)	0.10 (0.03 – 0.18)	0.07 (0.03 – 0.10)
C3	-	P5	0.11 (0.05 – 0.16)	0.07 (0.05 – 0.15)	0.08 (0.04 – 0.11)	0.08 (0.05 – 0.15)
C3	-	PO7	0.10 (0.03 – 0.20)	0.03 (0.01 – 0.21)	0.09 (0.05 – 0.15)	0.07 (0.03 – 0.11)
C3	-	PO3	0.08 (0.04 – 0.15)	0.10 (0.02 – 0.13)	0.07 (0.03 – 0.20)	0.07 (0.03 – 0.16)
C3	-	POz	0.07 (0.04 – 0.10)	0.08 (0.05 – 0.16)	0.10 (0.05 – 0.19)	0.08 (0.05 – 0.13)
C3	-	PO4	0.07 (0.04 – 0.10)	0.12 (0.06 – 0.18)	0.07 (0.05 – 0.09)	0.06 (0.03 – 0.09)
C3	-	PO8	0.04 (0.03 – 0.10)	0.11 (0.03 – 0.16)	0.04 (0.02 – 0.09)	0.05 (0.03 – 0.09)
C3	-	P6	0.09 (0.05 – 0.16)	0.09 (0.07 – 0.12)	0.05 (0.02 – 0.07)	0.05 (0.01 – 0.09)
C3	-	P2	0.03 (0.01 – 0.08)	0.09 (0.07 – 0.11)	0.11 (0.02 – 0.14)	0.06 (0.03 – 0.09)
C3	-	CPz	0.10 (0.08 – 0.14)	0.17 (0.08 – 0.20)	0.09 (0.02 – 0.23)	0.14 (0.07 – 0.19)
C3	-	CP4	0.05 (0.04 – 0.06)	0.08 (0.03 – 0.13)	0.07 (0.02 – 0.11)	0.07 (0.03 – 0.08)
C3	-	TP8	0.09 (0.03 – 0.15)	0.07 (0.04 – 0.16)	0.07 (0.04 – 0.11)	0.09 (0.04 – 0.13)

	C3 - C6	0.06 (0.05 – 0.08)	0.10 (0.05 – 0.12)	0.09 (0.04 – 0.13)	0.09 (0.04 – 0.12)
	C3 - C2	0.06 (0.04 – 0.13)	0.11 (0.03 – 0.19)	0.07 (0.04 – 0.12)	0.09 (0.04 – 0.11)
	C3 - FC4	0.06 (0.03 – 0.13)	0.11 (0.04 – 0.15)	0.13 (0.08 – 0.16)	0.10 (0.08 – 0.13)
	C3 - FT8	0.10 (0.06 – 0.16)	0.08 (0.05 – 0.18)	0.11 (0.06 – 0.15)	0.07 (0.02 – 0.09)
	C3 - F6	0.08 (0.05 – 0.11)	0.10 (0.08 – 0.14)	0.09 (0.05 – 0.14)	0.10 (0.03 – 0.16)
	C3 - AF8	0.06 (0.03 – 0.10)	0.09 (0.07 – 0.11)	0.05 (0.02 – 0.11)	0.08 (0.04 – 0.13)
	C3 - AF4	0.03 (0.02 – 0.06)	0.08 (0.05 – 0.13)	0.09 (0.04 – 0.15)	0.10 (0.05 – 0.15)
	C3 - F2	0.10 (0.07 – 0.13)	0.09 (0.04 – 0.14)	0.07 (0.05 – 0.21)	0.09 (0.04 – 0.25)
alpha	C3 - Fp1	0.16 (0.04 – 0.27)	0.09 (0.04 – 0.16)	0.19 (0.07 – 0.25)	0.15 (0.07 – 0.21)
	C3 - Fz	0.16 (0.10 – 0.21)	0.13 (0.07 – 0.18)	0.09 (0.03 – 0.18)	0.17 (0.10 – 0.21)
	C3 - F3	0.15 (0.07 – 0.19)	0.19 (0.14 – 0.25)	0.12 (0.05 – 0.22)	0.23 (0.14 – 0.28)
	C3 - F7	0.13 (0.06 – 0.19)	0.12 (0.05 – 0.18)	0.13 (0.07 – 0.18)	0.15 (0.02 – 0.19)
	C3 - FT9	0.19 (0.09 – 0.23)	0.12 (0.06 – 0.20)	0.14 (0.08 – 0.19)	0.19 (0.12 – 0.31)
	C3 - FC5	0.12 (0.08 – 0.17)	0.13 (0.11 – 0.26)	0.08 (0.05 – 0.21)	0.22 (0.19 – 0.27)

C3	-	FC1	0.20 (0.09 – 0.29)	0.22 (0.11 – 0.33)	0.18 (0.12 – 0.31)	0.34 (0.15 – 0.40)
C3	-	T7	0.10 (0.04 – 0.18)	0.15 (0.04 – 0.33)	0.21 (0.08 – 0.29)	0.15 (0.07 – 0.25)
C3	-	TP9	0.17 (0.09 – 0.21)	0.16 (0.08 – 0.34)	0.20 (0.09 – 0.32)	0.18 (0.07 – 0.26)
C3	-	CP5	0.27 (0.09 – 0.33)	0.24 (0.11 – 0.35)	0.22 (0.19 – 0.30)	0.22 (0.15 – 0.34)
C3	-	CP1	0.09 (0.03 – 0.35)	0.12 (0.07 – 0.36)	0.20 (0.05 – 0.26)	0.15 (0.10 – 0.20)
C3	-	Pz	0.15 (0.11 – 0.45)	0.11 (0.05 – 0.28)	0.28 (0.15 – 0.43)	0.18 (0.08 – 0.34)
C3	-	P3	0.43 (0.16 – 0.53)	0.28 (0.17 – 0.46)	0.33 (0.13 – 0.57)	0.48 (0.36 – 0.50)
C3	-	P7	0.22 (0.05 – 0.26)	0.20 (0.15 – 0.24)	0.25 (0.15 – 0.42)	0.34 (0.24 – 0.50)
C3	-	O1	0.09 (0.07 – 0.19)	0.17 (0.03 – 0.29)	0.21 (0.14 – 0.35)	0.21 (0.16 – 0.28)
C3	-	Oz	0.11 (0.08 – 0.22)	0.08 (0.03 – 0.21)	0.14 (0.09 – 0.21)	0.14 (0.07 – 0.23)
C3	-	O2	0.15 (0.12 – 0.19)	0.10 (0.08 – 0.19)	0.12 (0.04 – 0.21)	0.17 (0.11 – 0.23)
C3	-	P4	0.11 (0.08 – 0.31)	0.11 (0.06 – 0.23)	0.13 (0.03 – 0.36)	0.19 (0.02 – 0.43)
C3	-	P8	0.11 (0.06 – 0.25)	0.19 (0.09 – 0.27)	0.13 (0.09 – 0.28)	0.26 (0.15 – 0.34)
C3	-	TP10	0.15 (0.04 – 0.26)	0.10 (0.06 – 0.20)	0.09 (0.05 – 0.21)	0.17 (0.07 – 0.23)

C3	-	CP6	0.16 (0.07 – 0.22)	0.09 (0.04 – 0.11)	0.12 (0.07 – 0.16)	0.14 (0.08 – 0.37)
C3	-	CP2	0.09 (0.05 – 0.20)	0.18 (0.04 – 0.23)	0.18 (0.07 – 0.33)	0.11 (0.04 – 0.24)
C3	-	Cz	0.23 (0.09 – 0.41)	0.21 (0.13 – 0.28)	0.22 (0.14 – 0.33)	0.35 (0.25 – 0.50)
C3	-	C4	0.06 (0.04 – 0.19)	0.09 (0.06 – 0.15)	0.07 (0.04 – 0.18)	0.10 (0.04 – 0.18)
C3	-	T8	0.16 (0.07 – 0.20)	0.06 (0.05 – 0.13)	0.08 (0.04 – 0.15)	0.13 (0.09 – 0.26)
C3	-	FT10	0.11 (0.09 – 0.22)	0.15 (0.07 – 0.25)	0.18 (0.08 – 0.29)	0.17 (0.06 – 0.24)
C3	-	FC6	0.09 (0.03 – 0.17)	0.06 (0.03 – 0.19)	0.13 (0.02 – 0.19)	0.08 (0.06 – 0.21)
C3	-	FC2	0.11 (0.08 – 0.20)	0.17 (0.11 – 0.24)	0.15 (0.08 – 0.27)	0.19 (0.11 – 0.28)
C3	-	F4	0.09 (0.05 – 0.17)	0.05 (0.04 – 0.15)	0.07 (0.02 – 0.15)	0.09 (0.03 – 0.15)
C3	-	F8	0.12 (0.08 – 0.15)	0.17 (0.12 – 0.22)	0.10 (0.02 – 0.16)	0.16 (0.07 – 0.27)
C3	-	Fp2	0.15 (0.05 – 0.30)	0.12 (0.02 – 0.21)	0.08 (0.02 – 0.23)	0.14 (0.09 – 0.23)
C3	-	AF7	0.13 (0.09 – 0.20)	0.10 (0.06 – 0.20)	0.17 (0.10 – 0.32)	0.13 (0.08 – 0.18)
C3	-	AF3	0.10 (0.08 – 0.14)	0.10 (0.04 – 0.22)	0.09 (0.08 – 0.25)	0.20 (0.14 – 0.28)
C3	-	AFz	0.11 (0.08 – 0.15)	0.13 (0.04 – 0.21)	0.15 (0.03 – 0.20)	0.14 (0.09 – 0.28)

C3 - F1	0.16 (0.06 – 0.24)	0.13 (0.08 – 0.20)	0.12 (0.08 – 0.16)	0.19 (0.07 – 0.23)
C3 - F5	0.14 (0.09 – 0.19)	0.23 (0.06 – 0.32)	0.13 (0.09 – 0.18)	0.14 (0.07 – 0.21)
C3 - FT7	0.14 (0.11 – 0.18)	0.06 (0.04 – 0.19)	0.19 (0.05 – 0.23)	0.16 (0.02 – 0.29)
C3 - FC3	0.09 (0.04 – 0.18)	0.26 (0.11 – 0.32)	0.13 (0.04 – 0.19)	0.19 (0.10 – 0.29)
C3 - C1	0.21 (0.06 – 0.32)	0.25 (0.10 – 0.26)	0.22 (0.16 – 0.30)	0.35 (0.21 – 0.53)
C3 - C5	0.10 (0.04 – 0.19)	0.09 (0.05 – 0.23)	0.12 (0.05 – 0.31)	0.19 (0.08 – 0.27)
C3 - TP7	0.15 (0.08 – 0.23)	0.26 (0.10 – 0.35)	0.24 (0.07 – 0.31)	0.20 (0.09 – 0.34)
C3 - CP3	0.36 (0.15 – 0.48)	0.29 (0.17 – 0.36)	0.24 (0.13 – 0.40)	0.33 (0.18 – 0.41)
C3 - P1	0.28 (0.09 – 0.49)	0.19 (0.13 – 0.38)	0.31 (0.14 – 0.53)	0.33 (0.24 – 0.44)
C3 - P5	0.36 (0.21 – 0.41)	0.32 (0.24 – 0.39)	0.39 (0.18 – 0.54)	0.47 (0.37 – 0.54)
C3 - PO7	0.14 (0.09 – 0.25)	0.21 (0.15 – 0.35)	0.30 (0.09 – 0.47)	0.35 (0.19 – 0.47)
C3 - PO3	0.30 (0.09 – 0.39)	0.22 (0.15 – 0.45)	0.38 (0.13 – 0.57)	0.38 (0.30 – 0.46)
C3 - POz	0.24 (0.10 – 0.32)	0.17 (0.11 – 0.22)	0.27 (0.13 – 0.39)	0.18 (0.06 – 0.35)
C3 - PO4	0.13 (0.09 – 0.30)	0.09 (0.05 – 0.17)	0.17 (0.11 – 0.27)	0.16 (0.13 – 0.35)

C3	-	PO8	0.13 (0.05 – 0.32)	0.13 (0.07 – 0.22)	0.12 (0.10 – 0.22)	0.21 (0.16 – 0.31)
C3	-	P6	0.19 (0.11 – 0.25)	0.17 (0.04 – 0.24)	0.13 (0.10 – 0.20)	0.28 (0.21 – 0.36)
C3	-	P2	0.08 (0.06 – 0.35)	0.12 (0.04 – 0.20)	0.13 (0.05 – 0.40)	0.15 (0.02 – 0.30)
C3	-	CPz	0.14 (0.03 – 0.31)	0.11 (0.05 – 0.23)	0.14 (0.09 – 0.21)	0.09 (0.03 – 0.22)
C3	-	CP4	0.17 (0.10 – 0.25)	0.07 (0.04 – 0.21)	0.23 (0.05 – 0.33)	0.14 (0.07 – 0.22)
C3	-	TP8	0.09 (0.03 – 0.14)	0.13 (0.10 – 0.17)	0.11 (0.08 – 0.14)	0.18 (0.05 – 0.33)
C3	-	C6	0.12 (0.06 – 0.18)	0.09 (0.08 – 0.20)	0.10 (0.06 – 0.15)	0.16 (0.03 – 0.19)
C3	-	C2	0.09 (0.03 – 0.14)	0.12 (0.04 – 0.13)	0.10 (0.03 – 0.16)	0.13 (0.08 – 0.21)
C3	-	FC4	0.07 (0.04 – 0.11)	0.09 (0.08 – 0.15)	0.06 (0.04 – 0.16)	0.11 (0.05 – 0.19)
C3	-	FT8	0.12 (0.08 – 0.16)	0.15 (0.10 – 0.21)	0.11 (0.02 – 0.17)	0.16 (0.08 – 0.27)
C3	-	F6	0.12 (0.09 – 0.16)	0.13 (0.05 – 0.19)	0.06 (0.03 – 0.18)	0.09 (0.04 – 0.21)
C3	-	AF8	0.10 (0.04 – 0.27)	0.12 (0.07 – 0.17)	0.18 (0.08 – 0.25)	0.23 (0.16 – 0.31)
C3	-	AF4	0.08 (0.05 – 0.17)	0.14 (0.07 – 0.18)	0.10 (0.07 – 0.17)	0.20 (0.10 – 0.31)
C3	-	F2	0.13 (0.05 – 0.16)	0.11 (0.07 – 0.12)	0.05 (0.03 – 0.21)	0.10 (0.07 – 0.21)

beta				
C3 - Fp1	0.03 (0.01 – 0.05)	0.03 (0.02 – 0.05)	0.03 (0.03 – 0.07)	0.02 (0.01 – 0.05)
C3 - Fz	0.06 (0.05 – 0.07)	0.03 (0.01 – 0.06)	0.07 (0.04 – 0.09)	0.06 (0.03 – 0.09)
C3 - F3	0.02 (0.01 – 0.07)	0.03 (0.02 – 0.04)	0.03 (0.01 – 0.04)	0.05 (0.03 – 0.10)
C3 - F7	0.04 (0.02 – 0.05)	0.03 (0.01 – 0.06)	0.04 (0.03 – 0.05)	0.06 (0.03 – 0.09)
C3 - FT9	0.03 (0.02 – 0.06)	0.02 (0.01 – 0.05)	0.02 (0.01 – 0.03)	0.04 (0.02 – 0.08)
C3 - FC5	0.03 (0.02 – 0.04)	0.05 (0.02 – 0.06)	0.07 (0.04 – 0.13)	0.08 (0.05 – 0.14)
C3 - FC1	0.05 (0.04 – 0.09)	0.08 (0.03 – 0.13)	0.08 (0.04 – 0.12)	0.08 (0.03 – 0.14)
C3 - T7	0.09 (0.04 – 0.11)	0.08 (0.02 – 0.11)	0.07 (0.04 – 0.09)	0.05 (0.02 – 0.13)
C3 - TP9	0.04 (0.02 – 0.06)	0.06 (0.04 – 0.09)	0.05 (0.02 – 0.07)	0.06 (0.04 – 0.07)
C3 - CP5	0.16 (0.12 – 0.18)	0.04 (0.03 – 0.15)	0.09 (0.05 – 0.15)	0.13 (0.09 – 0.22)
C3 - CP1	0.06 (0.03 – 0.11)	0.06 (0.01 – 0.10)	0.08 (0.04 – 0.14)	0.06 (0.04 – 0.08)
C3 - Pz	0.03 (0.01 – 0.06)	0.04 (0.03 – 0.07)	0.04 (0.02 – 0.10)	0.03 (0.02 – 0.05)
C3 - P3	0.10 (0.07 – 0.11)	0.07 (0.03 – 0.11)	0.11 (0.05 – 0.14)	0.12 (0.07 – 0.19)
C3 - P7	0.15 (0.09 – 0.19)	0.09 (0.05 – 0.13)	0.12 (0.05 – 0.17)	0.11 (0.07 – 0.20)

C3	-	O1	0.03	(	0.01	–	0.06	)	0.04	(	0.02	–	0.07	)	0.04	(	0.02	–	0.09	)	0.03	(0.01 – 0.05)
C3	-	Oz	0.02	(	0.01	–	0.05	)	0.03	(	0.01	–	0.04	)	0.03	(	0.02	–	0.05	)	0.03	(0.01 – 0.06)
C3	-	O2	0.03	(	0.01	–	0.04	)	0.02	(	0.01	–	0.05	)	0.06	(	0.04	–	0.08	)	0.02	(0.01 – 0.04)
C3	-	P4	0.03	(	0.03	–	0.05	)	0.03	(	0.02	–	0.06	)	0.02	(	0.01	–	0.08	)	0.03	(0.02 – 0.04)
C3	-	P8	0.05	(	0.02	–	0.08	)	0.05	(	0.03	–	0.07	)	0.02	(	0.01	–	0.04	)	0.02	(0.02 – 0.04)
C3	-	TP10	0.02	(	0.01	–	0.03	)	0.02	(	0.02	–	0.04	)	0.04	(	0.01	–	0.06	)	0.03	(0.02 – 0.05)
C3	-	CP6	0.03	(	0.03	–	0.06	)	0.04	(	0.03	–	0.06	)	0.04	(	0.04	–	0.06	)	0.05	(0.03 – 0.10)
C3	-	CP2	0.02	(	0.02	–	0.04	)	0.03	(	0.01	–	0.05	)	0.03	(	0.01	–	0.03	)	0.04	(0.02 – 0.07)
C3	-	Cz	0.07	(	0.03	–	0.09	)	0.06	(	0.02	–	0.10	)	0.07	(	0.05	–	0.10	)	0.07	(0.05 – 0.15)
C3	-	C4	0.03	(	0.02	–	0.06	)	0.03	(	0.02	–	0.05	)	0.04	(	0.03	–	0.05	)	0.02	(0.02 – 0.06)
C3	-	T8	0.02	(	0.01	–	0.02	)	0.04	(	0.01	–	0.06	)	0.04	(	0.01	–	0.07	)	0.03	(0.02 – 0.05)
C3	-	FT10	0.03	(	0.02	–	0.05	)	0.02	(	0.01	–	0.06	)	0.02	(	0.02	–	0.03	)	0.04	(0.03 – 0.07)
C3	-	FC6	0.03	(	0.02	–	0.06	)	0.05	(	0.02	–	0.06	)	0.04	(	0.03	–	0.05	)	0.02	(0.01 – 0.05)
C3	-	FC2	0.04	(	0.02	–	0.06	)	0.04	(	0.03	–	0.07	)	0.04	(	0.03	–	0.07	)	0.03	(0.01 – 0.06)

C3	-	F4	0.05 (0.02 – 0.06)	0.04 (0.01 – 0.06)	0.02 (0.01 – 0.03)	0.05 (0.02 – 0.09)
C3	-	F8	0.04 (0.02 – 0.07)	0.03 (0.02 – 0.06)	0.02 (0.01 – 0.06)	0.03 (0.01 – 0.06)
C3	-	Fp2	0.04 (0.02 – 0.06)	0.03 (0.01 – 0.05)	0.05 (0.03 – 0.07)	0.03 (0.02 – 0.09)
C3	-	AF7	0.02 (0.02 – 0.04)	0.02 (0.01 – 0.06)	0.05 (0.03 – 0.09)	0.05 (0.03 – 0.08)
C3	-	AF3	0.05 (0.01 – 0.09)	0.03 (0.01 – 0.04)	0.04 (0.01 – 0.07)	0.06 (0.02 – 0.09)
C3	-	AFz	0.04 (0.02 – 0.05)	0.02 (0.01 – 0.04)	0.05 (0.02 – 0.06)	0.04 (0.02 – 0.07)
C3	-	F1	0.05 (0.03 – 0.08)	0.03 (0.01 – 0.05)	0.08 (0.02 – 0.11)	0.05 (0.03 – 0.09)
C3	-	F5	0.03 (0.02 – 0.04)	0.03 (0.02 – 0.07)	0.04 (0.03 – 0.05)	0.10 (0.04 – 0.13)
C3	-	FT7	0.07 (0.06 – 0.10)	0.05 (0.02 – 0.07)	0.04 (0.03 – 0.10)	0.06 (0.02 – 0.07)
C3	-	FC3	0.04 (0.02 – 0.07)	0.04 (0.01 – 0.07)	0.04 (0.02 – 0.07)	0.08 (0.03 – 0.14)
C3	-	C1	0.09 (0.05 – 0.14)	0.07 (0.04 – 0.12)	0.08 (0.03 – 0.11)	0.09 (0.06 – 0.15)
C3	-	C5	0.08 (0.06 – 0.14)	0.07 (0.06 – 0.09)	0.06 (0.03 – 0.07)	0.07 (0.04 – 0.11)
C3	-	TP7	0.12 (0.06 – 0.17)	0.07 (0.05 – 0.14)	0.08 (0.05 – 0.13)	0.09 (0.07 – 0.14)
C3	-	CP3	0.09 (0.06 – 0.13)	0.06 (0.05 – 0.09)	0.12 (0.07 – 0.15)	0.10 (0.05 – 0.18)

C3	-	P1	0.07 (0.05 – 0.08)	0.05 (0.03 – 0.08)	0.09 (0.03 – 0.10)	0.08 (0.03 – 0.10)
C3	-	P5	0.12 (0.09 – 0.19)	0.08 (0.04 – 0.14)	0.14 (0.03 – 0.16)	0.13 (0.11 – 0.23)
C3	-	PO7	0.09 (0.03 – 0.13)	0.06 (0.03 – 0.12)	0.10 (0.02 – 0.13)	0.07 (0.04 – 0.09)
C3	-	PO3	0.06 (0.03 – 0.10)	0.05 (0.02 – 0.10)	0.07 (0.02 – 0.13)	0.04 (0.02 – 0.07)
C3	-	POz	0.03 (0.02 – 0.04)	0.04 (0.02 – 0.05)	0.04 (0.02 – 0.05)	0.02 (0.01 – 0.04)
C3	-	PO4	0.03 (0.02 – 0.05)	0.03 (0.02 – 0.06)	0.04 (0.02 – 0.07)	0.02 (0.01 – 0.05)
C3	-	PO8	0.03 (0.02 – 0.05)	0.04 (0.02 – 0.04)	0.04 (0.02 – 0.07)	0.02 (0.01 – 0.04)
C3	-	P6	0.04 (0.02 – 0.06)	0.05 (0.02 – 0.06)	0.04 (0.01 – 0.07)	0.01 (0.01 – 0.04)
C3	-	P2	0.05 (0.02 – 0.07)	0.02 (0.01 – 0.04)	0.04 (0.03 – 0.06)	0.04 (0.03 – 0.04)
C3	-	CPz	0.03 (0.01 – 0.07)	0.05 (0.02 – 0.11)	0.07 (0.05 – 0.12)	0.06 (0.04 – 0.14)
C3	-	CP4	0.02 (0.01 – 0.03)	0.05 (0.04 – 0.06)	0.04 (0.02 – 0.08)	0.07 (0.04 – 0.08)
C3	-	TP8	0.02 (0.02 – 0.07)	0.05 (0.03 – 0.08)	0.04 (0.00 – 0.06)	0.05 (0.03 – 0.05)
C3	-	C6	0.02 (0.02 – 0.04)	0.02 (0.01 – 0.05)	0.04 (0.02 – 0.05)	0.02 (0.01 – 0.07)
C3	-	C2	0.02 (0.01 – 0.05)	0.03 (0.02 – 0.04)	0.03 (0.01 – 0.04)	0.02 (0.01 – 0.09)

C3	-	FC4	0.03 (0.02 – 0.05)	0.03 (0.02 – 0.07)	0.04 (0.02 – 0.04)	0.05 (0.02 – 0.06)
C3	-	FT8	0.04 (0.02 – 0.06)	0.02 (0.01 – 0.04)	0.03 (0.01 – 0.05)	0.03 (0.01 – 0.06)
C3	-	F6	0.05 (0.03 – 0.08)	0.04 (0.03 – 0.05)	0.03 (0.01 – 0.04)	0.04 (0.02 – 0.07)
C3	-	AF8	0.05 (0.04 – 0.09)	0.02 (0.01 – 0.04)	0.04 (0.03 – 0.07)	0.05 (0.02 – 0.07)
C3	-	AF4	0.04 (0.04 – 0.05)	0.02 (0.01 – 0.03)	0.04 (0.02 – 0.04)	0.03 (0.02 – 0.09)
C3	-	F2	0.06 (0.04 – 0.09)	0.04 (0.02 – 0.05)	0.03 (0.02 – 0.05)	0.05 (0.01 – 0.09)

Supplementary Table S2

The wPLI before and during the intervention for each frequency band.
